# ﻿Species diversity of freshwater glass eel (Anguilliformes, Anguillidae) of Yilan, Taiwan, with remark on two new records

**DOI:** 10.3897/zookeys.1220.125590

**Published:** 2024-12-09

**Authors:** Yen-Ting Lin, Yu-San Han

**Affiliations:** 1 Institute of Fisheries Science, College of Life Science, National Taiwan University, Taipei 10617, Taiwan National Taiwan University Taipei Taiwan

**Keywords:** *Anguillaborneensis*, *Anguillainterioris*, glass eel, new records

## Abstract

Yilan, Taiwan is the first place in East Asia where freshwater glass eels, the juvenile stage of *Anguilla* species, arrive by ocean currents. We collected glass eels by fyke net in Lanyang River estuary twice a month from July 2010 to November 2023. By morphological examination and sequencing of the mitochondrial cytochrome b gene, we identified seven species of *Anguilla*. Most of the glass eels captured in Yilan belonged to the species *A.japonica*, *A.marmorata*, and *A.bicolorpacifica*. Only a few were *A.luzonensis*, and two *A.celebesensis* were recorded. In addition, two species were recorded for the time time from Taiwan; *A.interioris* and *A.borneensis* were confirmed by cytochrome b sequencing. Thus, we increase the number of *Anguilla* species in Taiwan from five to seven.

## ﻿Introduction

The freshwater eel (*Anguilla* spp.) comprises 16 species and three subspecies (Arai 2016a). All *Anguilla* species are catadromous fish, meaning they migrate to the ocean to spawn (Arai and Chino 2012). The leaf-like larvae of *Anguilla* species, known as leptocephali, are carried by ocean currents and undergo metamorphosis into eel-like juveniles, which are known as glass eels (Tsukamoto et al. 2002; Hatakeyama et al. 2022). Glass-eel fishing is crucial for the eel aquaculture industry, as there are no artificial reproduction techniques for commercial purposes (Okamura et al. 2014). In Taiwan, the dispersal of glass eels is primarily influenced by the Kuroshio Current (Hsiung et al. 2022b). Yilan, Taiwan, is renowned as the largest glass-eel fishing ground in Taiwan due to its proximity to the Kuroshio. Notably, Yilan holds the distinction of being the first location in East Asia where glass eels arrive, establishing it as a significant hub for this crucial stage in the eel life cycle (Han et al. 2016a).

To date, five *Anguilla* species have been identified and recorded in Taiwan (Leander et al. 2012; Han et al. 2016b). Among these, *A.japonica*, *A.marmorata*, and *A.bicolorpacifica* are the most prevalent species (Han 2001; Hsu et al. 2019), while *A.luzonensis* and *A.celebesensis* are notably very rare and primarily observed as glass eel in Taiwan (Teng et al. 2009; Han et al. 2016b). Previous studies suggest that *A.japonica*, *A.marmorata*, and *A.bicolorpacifica* share a common spawning area near the southern West Mariana Ridge (Kuroki et al. 2009; Arai 2016b), whereas other tropical eel species (*A.celebesensis*, *A.borneensis*, *A.luzonensis*, and *A.interioris*) have been identified near southern Mindanao Island as their spawning grounds (Aoyama et al. 2003; Wouthuyzen et al. 2009; Arai 2014, 2016b). Due to the morphological challenges in distinguishing tropical eel glass eels (Minegishi et al. 2005), DNA barcoding techniques, as highlighted by Wibowo et al. (2021), provide a precise method for the identification of species. Previous research also indicates that mitochondrial cytochrome b gene fragments are suitable for the identification of freshwater eels (Han et al. 2008). This study aims to analyse glass-eel samples captured in Yilan from July 2010 to November 2023. Through DNA sequencing, the goal is to confirm the number of freshwater glass-eel species transported to Taiwan during this period.

## ﻿Materials and methods

### ﻿Sample collection

Glass eels were collected twice a month at night using a fyke net positioned in the estuary of the Yilan River (24.7162°N, 121.8352°E) from July 2010 to November 2023. Following the capture, all the samples were immersed in a 95% ethanol solution for measurement and preservation. All freshwater glass-eel specimens were deposited in the Institute of Fisheries, National Taiwan University (**NTUIFS**). Recent research adhered to ethical regulations set forth by the Institutional Animal Care and Use Committee (IACUC) under approval number NTU-110-EL-00152.

### ﻿Morphological measurement

The method for morphological identification of anguillid glass eels was adapted from Han et al. (2012), and the description of the pigmentation stage followed Fukuda et al. (2013). Four morphological parameters were measured using digital callipers with an accuracy of 0.1 mm: total length (TL), head length (HL), pre-dorsal length (PDL), and pre-anal length (PAL). The fin-difference ratio was then calculated using the formula shown below. Glass eels with fin differences exceeding 13% in Yilan were consistently identified as *A.marmorata* (Han et al. 2012). Therefore, specimens displaying black pigment on the tail and fin differences <13% were chosen for mitochondrial cytochrome b gene sequencing.

$\text{ Fin Difference Ratio }(\%)=\frac{PAL(\;mm)-PDL(\;mm)}{TL(\;mm)}\times 100$

### ﻿Mitochondrial cytochrome b gene sequencing

Freshwater glass-eel specimens with a fin-difference ratio <13% were DNA sequenced for precise identification; these amounted to 281 samples. Genomic DNA was extracted from the dorsal-fin tissue of the glass eels using the FavorPrep Tissue Genomic DNA Extraction Mini Kit (Favorgen, Taiwan). Polymerase chain reaction (PCR) was carried out to amplify a segment of mitochondrial cytochrome b using forward primer: cytb-F (5′-GAT GCC CTA GTG GAT CTA CC-3′) and reverse primer: cytb-R (5′-TAT GGG TGT TCT ACT GGT AT-3′), which was adapted from Han et al. (2008). The resulting PCR product (approximately 1000 bp) was sequenced using the primers cytb-F or cytb-R (by Genomic Biotech Inc., Taiwan), following protocols from Han et al. (2008). Sequencing results were submitted to the National Center for Biotechnology Information (**NCBI**) GenBank (https://blast.ncbi.nlm.nih.gov/Blast.cgi) to confirm species.

## ﻿Results

### ﻿Diversity of freshwater glass eel

A total of 29,442 freshwater glass eels were collected between July 2010 and November 2023. The composition of freshwater glass-eel species is shown in Table 1. *Anguillajaponica* and *A.marmorata* were the most prevalent species, comprising 95.4% of our captures (Table 1). Although *A.bicolorpacifica* and *A.luzonensis* were infrequently captured, they still represented 3.9% and 0.7% of all specimens, respectively, and two *A.celebesensis* were also recorded (Table 1). Additionally, two species were found in Taiwan for the first time: *A.interioris* and *A.borneensis* (Table 1).

**Table 1.** Number of species (*n*) and percentage contributions of freshwater glass eels collected in Yilan.

Species	*n*	Percentage contributions
*A.japonica*	14217	48.3
*A.marmorata*	13864	47.1
*A.bicolorpacifica*	1152	3.9
*A.luzonensis*	205	0.7
*A.celebesensis*	2	<0.01
*A.interioris*	1	<0.01
*A.borneensis*	1	<0.01

### ﻿New *Anguilla* records from Taiwan

The sequencing results of the two new records, total two specimens (NTUIFS IL13’0812-76 and NTUIFS IL21’0715-207), and the best matched BLAST results are shown in Table 2.

**Table 2.** Sequencing BLAST results of three new records freshwater glass eel.

Specimen	Species	Percent identity (%)	NCBI accession
NTUIFS IL13’0812-76	*Anguillainterioris*	99.4	HG965574.1
NTUIFS IL21’0715-207	*Anguillaborneensis*	99.4	NC_006536.1*

*NC_006536.1 was identified as *A.malgumora*, which is a junior synonym of *A.borneensis* according to Minegishi et al. (2005).

#### ﻿Family Anguillidae

##### ﻿ Anguilla interioris

Taxon classificationAnimaliaAnguilliformesAnguillidae

Whitley, 1938

B5F9AE0C-0F36-5D52-AC73-EFCA6232C2D7

**Figure 1.**
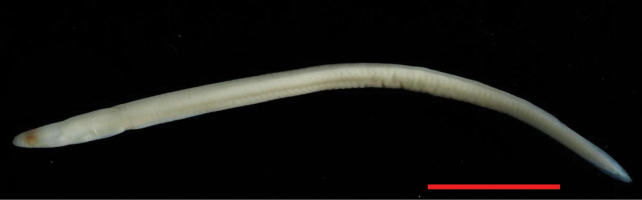
*Anguillainterioris* (NTUIFS IL13’0812-76), 46 mm TL. Preserved in 95% alcohol. Scale bar: 10 mm.

**Figure 2.**
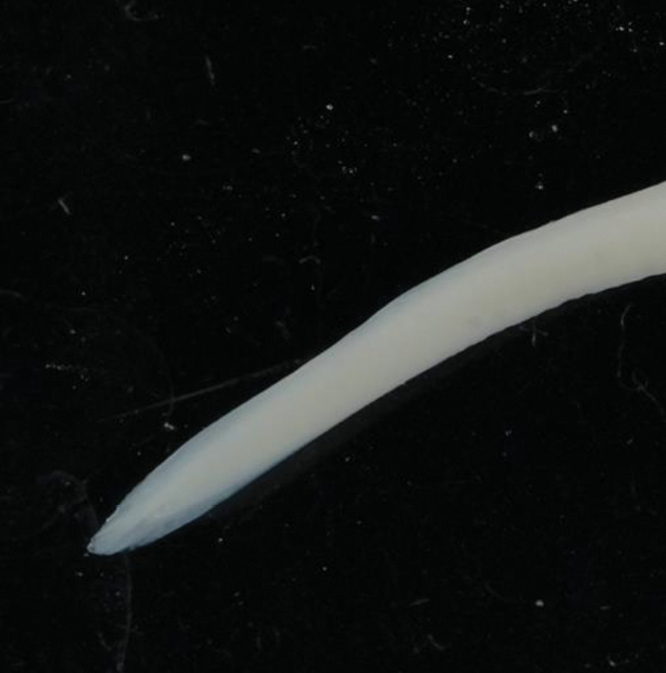
Pigmentation on the tail tip of *Anguillainterioris* (NTUIFS IL13’0812-76).

Figs 1 , 2 , Table 3

###### Material examined.

NTUIFS IL13’0812-76, 46 mm TL, off the estuary of the Yilan River, Yilan, northeastern Taiwan (24.7162°N, 121.8352°E), 12 August 2013, fyke net, collected by Yu-San Han.

###### Short description.

PDL 29.3% in TL; PAL 39.1% in TL; fin-difference ratio 9.78%. Body elongate, head length 13.1% TL. The specimen was in fresh condition, with black pigment distributed on the caudal fin and slightly on the caudal peduncle; pigmentation stages V_B2_ (Fig. 2).

###### Distribution.

New Guinea (Aoyama et al. 2000); Philippines (glass eel only, Wibowo et al. 2021); Indonesia (leptocephalus only, Kuroki et al. 2006; all stages, Zan et al. 2022); Taiwan (glass eel only, present study).

**Table 3.** The morphological parameters of seven freshwater glass eel collected in Yilan.

Species	TL (mm)	PDL (mm)	PAL (mm)	Fin-difference ratio (%)
*A.japonica*	61.1±2.5	15.1±0.9	20.1±0.7	9.2±1.3
*A.marmorata*	51.4±2.7	11.8±0.8	19.4±1.1	15.5±0.8
*A.bicolorpacifica*	49.2±2.3	18.3±1.6	18.5±1.6	0.5±0.5
*A.luzonensis*	52.9±2.7	13.7±0.7	19.4±1.0	11.4±1.1
*A.celebesensis*	45.3	12.8	17.5	10.4
*A.interioris*	46.0	13.5	18.0	10.1
*A.borneensis*	49.5	13.0	18.0	9.8

###### Remarks.

The distribution of *A.interioris* has been primarily known from only New Guinea (Aoyama et al. 2000). However, a study by Kuroki et al. (2006) documented the leptocephalus of *A.interioris* in the Indonesian Archipelago, marking the first expansion of the species beyond its then-known range. Additionally, records of *A.interioris* have been identified using DNA sequencing from Indonesia and southern Mindanao, Philippines (Wibowo et al. 2021; Zan et al. 2022). Herein, we present the first record of *A.interioris* glass eel from Taiwan.

##### ﻿ Anguilla borneensis

Taxon classificationAnimaliaAnguilliformesAnguillidae

Popta, 1924

F5EF67D7-5E31-5D96-B050-2CD84C28E5CB

**Figure 3.**
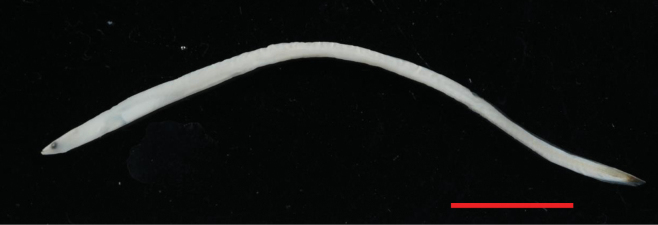
*Anguillaborneensis* (NTUIFS IL21’0715-207), 49.5mm TL. Preserved in 95% alcohol. Scale bar: 10 mm.

**Figure 4.**
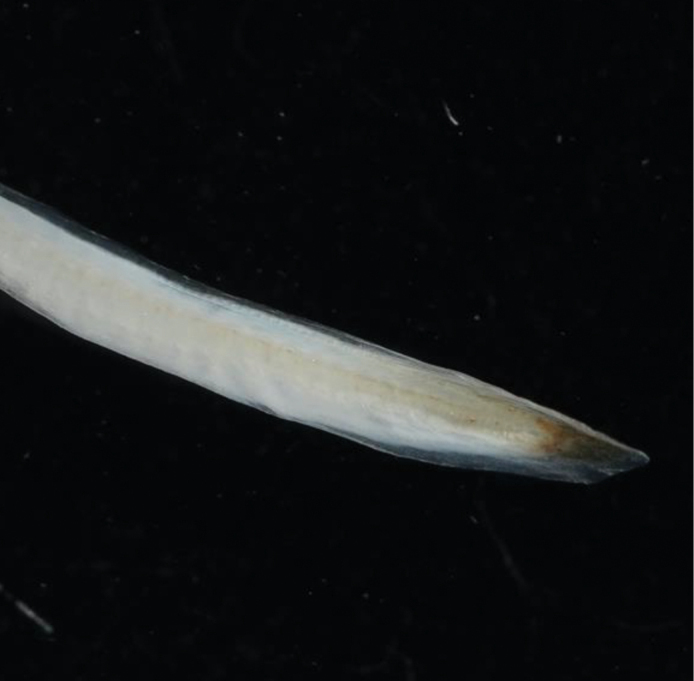
Pigmentation on the tail tip of *Anguillaborneensis* (NTUIFS IL21’0715-207).

Figs 3 , 4 , Table 3

###### Material examined.

NTUIFS IL21’0715-207, 49.5 mm TL, off the estuary of the Yilan River, Yilan, northeastern Taiwan (24.7162°N, 121.8352°E), 15 July 2021, fyke net, collected by Yen-Ting Lin.

###### Short description.

PDL 26.3% in TL; PAL 36.4% in TL; fin-difference ratio 10.1%. Body extremely elongate, head length 10.1% TL. The specimen was in fresh condition, with black pigment distributed on the caudal peduncle and caudal fin; pigmentation stages V_A_ (Fig. 4).

###### Distribution.

Indonesia (Watanabe et al. 2014); Taiwan (glass eel only, present study).

###### Remarks.

The best-matched GenBank accession number for NTUIFS IL21’0715-207 was found to be NC_006536.1, which corresponds to *A.malgumora* submitted by Minegishi et al. (2005). However, it is noteworthy that *A.malgumora* was identified as a junior synonym of *A.borneensis* by Minegishi et al. (2005). Based on the comprehensive examination by Minegishi et al. (2005) and the detailed description provided in NC_006536.1, we can confidently affirm that our specimen NTUIFS IL21’0715-207 is *A.borneensis*. Herein, we report the first record of *A.borneensis* outside of the Indonesia (Watanabe et al. 2014).

## ﻿Discussion and conclusion

Leptocephali and glass eels primarily rely on ocean currents for transport (Kuroki et al. 2016). In Taiwan, the main current responsible for transporting glass eels is the North Equatorial Current (NEC), followed by the Kuroshio, which is known to carry the most abundant anguillid species (*A.japonica* and *A.marmorata*) to the region (Hsiung et al. 2022a). Additionally, other tropical eels (*A.bicolorpacifica*, *A.luzonensis*, and *A.celebesensis*) may reach Taiwan via the bifurcation region of the NEC near the Philippine coast, which could potentially transport glass eels from southern Mindanao Island to the Kuroshio (Aoyama et al. 2015; Rudnick et al. 2015). The two species identified in our study align with previous research on the diversity of tropical glass eels (*A.celebesensis*, *A.interioris*, and *A.borneensis*) in southern Mindanao (Shirotori et al. 2016).

Previous research based on differences in Sr:Ca ratios in the leptocephalus otoliths has shown the presence of two populations of *A.interioris*, with one population in the Indian Ocean and another in the Pacific Ocean (Kuroki et al. 2006). Furthermore, leptocephali of the Pacific Ocean population of *A.interioris* potentially are transported to Taiwan via the Mindanao Current which ultimately forms a connection with the Kuroshio and the Mindanao Eddy (Kuroki et al. 2006).

The distribution of leptocephali and glass eels of the Indonesian *A.borneensis*, which is considered the most basal *Anguilla* species, remains unclear (Aoyama et al. 2001). The spawning area of *A.borneensis* may overlap with other basal tropical eels (*A.celebesensis*, *A.interioris*, *A.marmorata*, and *A.bicolorbicolor*) in Indonesia in the western Pacific Ocean (Arai and Abdul Kadir 2017); this suggests the possibility that a similar pathway to Taiwan is followed, as by *A.interioris* and *A.celebesensis* (Han et al. 2016b).

Alternatively, it is possible that if *A.borneensis* and *A.interioris* establish a new population in the western Pacific Ocean, their larvae could be carried to Taiwan via the North Equatorial Current (NEC) and the Kuroshio. Additionally, some alien freshwater eel species have escaped from aquaculture ponds and have been reported to have similar migration behaviour of native eel in East Asia (Okamura et al. 2002). Examples include *A.rostrata*, which has been discovered in Taiwanese waters (Han et al. 2002), and the European eel, *A.anguilla*, which was captured in the East China Sea and Japanese waters (Aoyama 2000; Okamura et al. 2002). Therefore, the possibility of alien eel species establishing new populations in the West Pacific Ocean cannot be discounted, whether caused by human activities (*A.rostrata* and *A.anguilla*) or by natural phenomenon (*A.interioris* and *A.borneensis*) (Aoyama 2000; Han et al. 2002).

Although there are seven species of freshwater glass eel recorded in Taiwan, only elvers of *A.japonica*, *A.marmorata*, *A.luzonensis*, and *A.bicolorpacifica* had been found in streams (Tzeng and Tabeta 1983; Watanabe et al. 2013; Hsu et al. 2019). The existence of the elvers and adults of *A.celebesensis*, *A.interioris*, and *A.borneensis* still need confirmation in the field.

In conclusion, the present study increases the number of freshwater glass-eel species in Taiwan from five to seven (Leander et al. 2012; Han et al. 2016b), with the addition two new species records in this paper.

## Supplementary Material

XML Treatment for Anguilla interioris

XML Treatment for Anguilla borneensis

## ﻿Additional information

### Data availability

All of the data that support the findings of this study are available in the main text or Supplementary Information.

## Supplementary materials

Supplementary material 1 NTUIFS IL13'0812-76 sequencing data Data type txt Explanation note The sequencing results of NTUIFS IL13'0812-76. https://binary.pensoft.net/file/1192303This dataset is made available under the Open Database License (http://opendatacommons.org/licenses/odbl/1.0/). The Open Database License (ODbL) is a license agreement intended to allow users to freely share, modify, and use this Dataset while maintaining this same freedom for others, provided that the original source and author(s) are credited.Yen-Ting Lin, Yu-San Han

Supplementary material 2 NTUIFS IL21'0715-207 sequencing data Data type txt Explanation note The sequencing results of NTUIFS IL21'0715-207. https://binary.pensoft.net/file/1192304This dataset is made available under the Open Database License (http://opendatacommons.org/licenses/odbl/1.0/). The Open Database License (ODbL) is a license agreement intended to allow users to freely share, modify, and use this Dataset while maintaining this same freedom for others, provided that the original source and author(s) are credited.Yen-Ting Lin, Yu-San Han
